# Systematics and distribution of *Cristaria plicata* (Bivalvia, Unionidae) from the Russian Far East

**DOI:** 10.3897/zookeys.580.7588

**Published:** 2016-04-12

**Authors:** Olga K. Klishko, Manuel Lopes-Lima, Elsa Froufe, Arthur E. Bogan, Vera Y. Abakumova

**Affiliations:** 1 Institute of Natural Resources, Ecology and Cryology, Russian Academy of Sciences Siberian Branch, Chita 672014, Russia; 2 Interdisciplinary Centre of Marine and Environmental Research (CIIMAR/CIMAR), University of Porto, Rua dos Bragas 289, 4050-123 Porto, Portugal; 3 Mollusc Specialist Group, Species Survival Commission, International Union for Conservation of Nature (SSC/IUCN), c/o IUCN, 219 Huntington Road, Cambridge, United Kingdom; 4 Research Laboratory, North Carolina Museum of Natural Sciences, MSC 1626, Raleigh, NC 27699-1626, United States of America

**Keywords:** Bivalvia, Unionidae, Anodontini, COI, morphometry, Russia

## Abstract

The number of anodontine bivalve species placed in the genus *Cristaria* (Bivalvia, Unionidae) from the Russian Far East is still not stable among authors. Some recognize only one valid species *Cristaria plicata* (Leach, 1815) while others accept two additional species, *Cristaria tuberculata* Schumacher, 1817 and *Cristaria herculea* (Middendorff, 1847). In the present study, these taxonomic doubts are addressed using analyses of mitochondrial DNA sequences and shell morphometry. No significant differences have been revealed by the COI DNA sequences or the main statistical morphometric indices from the three *Cristaria* forms. In the specimens analysed, changes in shell morphometry with age suggest that original descriptions of the different forms may be attributed solely to differences in age and sex. We consider that *Cristaria plicata*, *Cristaria tuberculata* and *Cristaria herculea* from the Russian Far East should be considered as a single species, namely *Cristaria plicata* (Leach, 1815), with *Cristaria tuberculata* and *Cristaria herculea* as junior synonyms. The geographic range of *Cristaria plicata* and its conservation status are also presented here.

## Introduction

It is well known that freshwater bivalves of the Unionidae provide important ecosystem functions (Vaughn and Hakenkamp 2001; Aldridge, Fayle and Jackson 2007) and services to humans (Lopes-Lima et al. 2014). However, they are among the most threatened groups worldwide and many of their populations are in decline (Bogan 1993; Klishko 2012; Lopes-Lima et al. 2014). Due to this fact, studies of the diversity of these taxa, at and below the species level, are urgently required for effective conservation.

The different classification systems used in Russia considerably hamper identification of species and inventories of the molluscan fauna. This is the situation for the taxonomy of freshwater bivalve species of the genus *Cristaria* from the Russian Far East, which has been contentious among taxonomists. According to the classification system of Zhadin (1938, 1952, 1965), *Cristaria plicata* (Leach, 1815) was the sole representative of the genus *Cristaria* in Russia. Similarly, other international authors have also synonymized most of the described forms of *Cristaria*, including all Russian species, under the type species *Cristaria plicata*, present in Eastern Asia, from Russia to Japan, South Korea, China and Indochina (Haas 1969; Brandt 1974; Đặng et al. 1980; Kondo 2008; He and Zhuang 2013; Klishko et al. 2014). Nevertheless, some authors still recognize two separate species in Far East Russia, *Cristaria herculea* (Middendorff, 1847) and *Cristaria tuberculata* Schumacher, 1817, or even three species: *Cristaria herculea*, *Cristaria tuberculata*, and *Cristaria plicata* (Sayenko et al. 2005).

Using a conchological classification system, some Far East Russian specimens from the collection of the Zoological Institute Museum, St. Petersburg (ZIM-SP) have been attributed only to *Cristaria tuberculata* Schumacher, 1817 and indicated as the type species of the genus (Schumacher 1817; Moskvicheva 1973; Zatravkin and Bogatov 1987). Specimens of *Cristaria plicata* from Khanka Lake identified by Zhadin in 1927 and Starobogatov in 1967 (specimens from the ZIM-SP collection) were among these. Since then, *Cristaria plicata* has disappeared from the literature on the East Russian fauna and *Cristaria tuberculata* Schumacher, 1817 and *Cristaria herculea* (Middendorff, 1848) were the only species recognized in the region (Zatravkin and Bogatov 1987; Prozorova and Sayenko 2001; Starobogatov et al. 2004).

Only one conchological character, shell convexity, was used to separate both species. *Cristaria herculea*, with laterally compressed shells, is widespread in the Amur River basin and Khanka Lake in Russia, as well as the Buyr-Nor Lake in Mongolia. *Cristaria tuberculata*, with inflated shells, is rare and limited to the Russian Far East, in Khanka Lake and the Ussury River basin (Moskvicheva 1973; Zatravkin and Bogatov 1987; Prozorova and Sayenko 2001; Starobogatov et al. 2004). However, there is increasing evidence that suggests the existence of a single *Cristaria* species in Far East Russia.

Based on conchological observations, Graf (2007) and He and Zhuang (2013) and on the electrophoretic myogen spectra (Kodolova and Logvinenko 1987, 1988) ascertained that *Cristaria tuberculata* and *Cristaria herculea* are synonyms of *Cristaria plicata*. Additionally, studies on the reproductive cycles (Higashi and Hayashi 1964; Prozorova and Sayenko 2001) and glochidial characteristics of *Cristaria herculea* and *Cristaria tuberculata* from the Russian Far East and of *Cristaria plicata* from Japan and China also revealed no significant differences among these forms (Inaba 1941, 1964; Wu et al. 2000; Sayenko 2006). Finally, the recent publication on *Cristaria herculea* from the Transbaikalia, using morphological, anatomical and molecular data presented convincing arguments that *Cristaria herculea* should be synonymized with *Cristaria plicata* (Klishko et al. 2014).

Despite all this evidence, these two species continue to be recognized as independent species by the Russian system of taxonomy (Starobogatov et al. 2004). Therefore, it is necessary to integrate and gather conclusive evidence for the *Cristaria* species identification, including additional conchological, anatomical and molecular characters.

The main goals of the present work are to establish the taxonomic status and phylogenetic relationships of *Cristaria tuberculata* and *Cristaria herculea* from the Russian Far East, and *Cristaria plicata* from the adjacent territories of Transbaikalia and China. This will be achieved by using molecular analysis of the Cytochrome *c* Oxidase I gene fragment and morphometric statistical analysis of the shells. Finally, the distributional range of these taxa in Russia and Eastern Asia will also be evaluated.

## Material and methods

### Genetic analyses

For molecular analyses, *Cristaria tuberculata* specimens were collected in 2014 from the Luchegorsky Reservoir, of Ussury River Basin, in Russian Far East. *Cristaria herculea* specimens from Khanka Lake, Primorye, were retrieved from the collection of the Institute of Biology and Soil Science, Far East Branch, Russian Academy of Sciences (Vladivostok). Whole genomic DNA was extracted from small tissue pieces of 2 *Cristaria tuberculata* and 2 *Cristaria herculea* individuals (Table 1), using a standard high-salt protocol (Sambrook et al. 1989). PCR and sequencing conditions are described in Froufe et al. (2014). Forward and reverse sequences were edited and assembled using CHROMASPRO 1.7.4 (Technelysium, Tewantin, Australia) and all sequences were then aligned with CLUSTALW, in BIOEDIT 7.2.5 (Hall 1999). For a preliminary analysis, all *Cristaria* sp. CO1 sequences available on GenBank were downloaded (n = 65). Afterwards, 52 of these sequences were excluded from the present analyses for clarity (they all represented different haplotypes that fell inside the *Cristaria plicata* clade, see results; data not shown). A final alignment was analysed, where the selected outgroups included one *Anodonta beringiana* individual and one *Sinanodonta woodiana* (Table 1). This final alignment included 21 individuals in total, with the two *Cristaria herculea* sequences used from Klishko et al. (2014) and the four newly sequenced individuals. The best-fit model of nucleotide substitution evolution under corrected Akaike Information Criterion was estimated using JMODELTEST 2.1.4 (Darriba et al. 2012). Model HKY+I was chosen and used in the phylogenetic analysis. Phylogenetic Bayesian Inference (BI) was performed using MRBAYES version 3.1.2 (Ronquest and Huelsenbeck 2003). Two independent runs with 24 million generations long were sampled at intervals of 1,000 generations, producing a total of 24,000 trees. Burnin was determined upon convergence of log likelihood and parameters estimation values using TRACER 1.4 (Rambaut and Drummond 2007). Estimates of sequence divergence (uncorrected *p*-distances) were assessed using MEGA 6.06 software (Tamura et al. 2013).

**Table 1.** List of specimen samples sequenced (CO1) and GenBank accession numbers. *Unpublished

Species	Locality	Country	Code/GenBank	Study
*Cristaria tuberculata*	Luchegorsky reservoir	Russia	Biv1530/KT348507	This study
*Cristaria tuberculata*	Luchegorsky reservoir	Russia	Biv1531/KT348508	This study
*Cristaria herculea*	Onon River	Russia	Biv246/KT362704	Klishko et al. (2014)
*Cristaria herculea*	Charanorsky Reservoir	Russia	Biv247/KT362705	Klishko et al. (2014)
*Cristaria herculea*	Khanka Lake	Russia	Biv1537a/KU297678	This study
*Cristaria herculea*	Khanka Lake	Russia	Biv1537b/KU297678	This study
*Cristaria plicata*	Lower Yangtze	China	EU698893; EU698897; EU698913; EU698948	Jia and Li*
*Cristaria plicata*	Unknown	China	JF700152; JF700153	Zhang et al.*
*Cristaria plicata*	Zhejiang	China	FJ986302	Jiang et al. (2010)
*Cristaria plicata*	Unknown	South Korea	GQ451860	Park et al.*
*Cristaria plicata*	Unknown	South Korea	GU944476	Lee et al. (2012)
*Cristaria* sp.	Lower Yangtze	China	EU698909; EU698910; EU698940; EU698942	Jia and Li*
*Anodonta beringiana*	Jo-Jo Lake	Canada	DQ272370	Gustafson and Iwamoto (2005)
*Sinanodonta woodiana*	Unknown	Poland	HQ283347	Soroka and Burzynski*

### Morphometric analyses

For the *Cristaria herculea* and *Cristaria tuberculata* morphometric analyses, specimens of *Cristaria* from the collections of the Institute of Natural Resources, Ecology and Cryology, of the Russian Academy of Sciences Siberian Branch (INREC-RAS-SB) and from the Zoological Institute Museum, St. Petersburg (ZIM-SP), including the specimens used for the original species descriptions (Zhadin 1938, 1952; Moskvicheva 1973; Zatravkin and Bogatov 1987) were measured. In addition, 20 shells of *Cristaria plicata* from Khanka Lake of the same series, identified by Zhadin in 1927 and Starobogatov in 1967 were also measured and included in the analyses. The total shell length (L), maximal shell inflation (B) and shell height at umbo (H) were measured to the nearest 0.1 mm. Furthermore, the distance from the umbo to the end of the posterior end of the lateral tooth was also measured, in order to calculate the ratio of maximal shell inflation to this distance. This parameter is used to separate two species of *Cristaria* from Eastern Russia, according to the identification keys of Zatravkin and Bogatov (1987) and Starobogatov et al. (2004). This ratio is herein designated as the R-index. According to the published identification keys, the R-index for *Cristaria herculea* should be less than 0.82, while for *Cristaria tuberculata* it should to be higher than 0.85. Standard morphometric shell indices, namely the ratio of shell inflation to shell height (B/H), ratio of shell inflation to shell length (B/L), and ratio of shell height to shell length (H/L) were calculated. The poorly expressed morphological discreteness between species was examined using a discriminant analysis based on a linear combination of the three morphometric indexes - use of ratios provided independence from shell size. The reliability of discreteness between species was assessed by λ (Wilk’s lambda) value. This may vary from 0 to 1 where λ = 0 indicates ideal discriminatory power of the morphometric predictors and λ = 1 indicates no discriminatory ability of the model. Statistical analyses were made using MICROSOFT EXCEL 2010 and STATISTICA v.6.1 software.

### Geographic distribution

The distribution of *Cristaria* taxa in Far Eastern Russia and adjacent territories was compiled using data from the INREC-RAS-SB and ZIM-SP collections, and from an extensive bibliographic search (Suppl. material 1). The locations of *Cristaria plicata* in the Upper Amur River Basin and Buyr-Nor Lake, Mongolia, were georeferenced to a precision of ± 0.2 km. However, the locations of *Cristaria* taxa in the Middle Amur River Basin and remaining territory recovered from the literature and from the labels of the ZIM-SP collections have less accurate localities (Suppl. material 1).

## Results

### Genetic analyses

**Figure 1.**
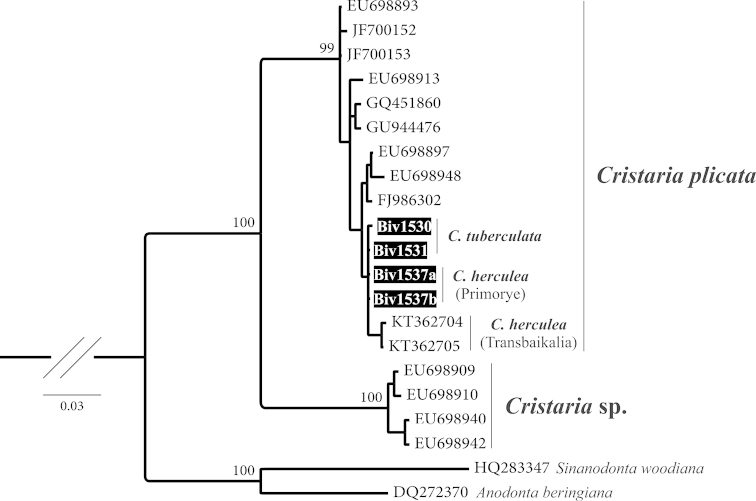
Phylogenetic tree obtained by Bayesian Inference analysis, using mtDNA fragments (CO1). Support values are given as Bayesian posterior probability above nodes, except for those within major clades, which have been omitted for clarity. Available sequences downloaded from GenBank and new sequences codes refer to Table 1.

Three haplotypes were retrieved from the four newly sequenced individuals: two in *Cristaria tuberculata* specimens (i.e., Biv1530 and Biv1531; Fig. 1), and one in *Cristaria herculea* (i.e., Biv1537a and Biv1537b; Fig. 1). The aligned CO1 sequences had a total length of 618 bp, with 141 polymorphic and 90 parsimony informative sites. No indels and no unexpected stop codons were observed after translating all sequences to amino acids. The tree topology resulting from the BI analyses is shown in Fig. 1. Two major mtDNA clades were retrieved with strong support: one includes all individuals from *Cristaria plicata* together with the four new individuals sequenced for this work (uncorrected *p*-distance among them <1.1%), and the other clade includes six individuals, also originally assigned to *Cristaria plicata* (Jia and Li, Unpublished). Therefore, the newly sequenced individuals morphologically identified as *Cristaria tuberculata* (Biv1530 and Biv1531; Fig. 1) and *Cristaria herculea* (Biv1537a and Biv1537b; Fig. 1) cluster within *Cristaria plicata*. As already noted by Klishko et al. (2014), the phylogeny of the genus *Cristaria* in China needs further evaluation, since the uncorrected *p*-distance of 9.9% between the two retrieved clades strongly suggests the existence of two different *Cristaria* species in this data set.

### Morphometric analyses

**Figure 2.**
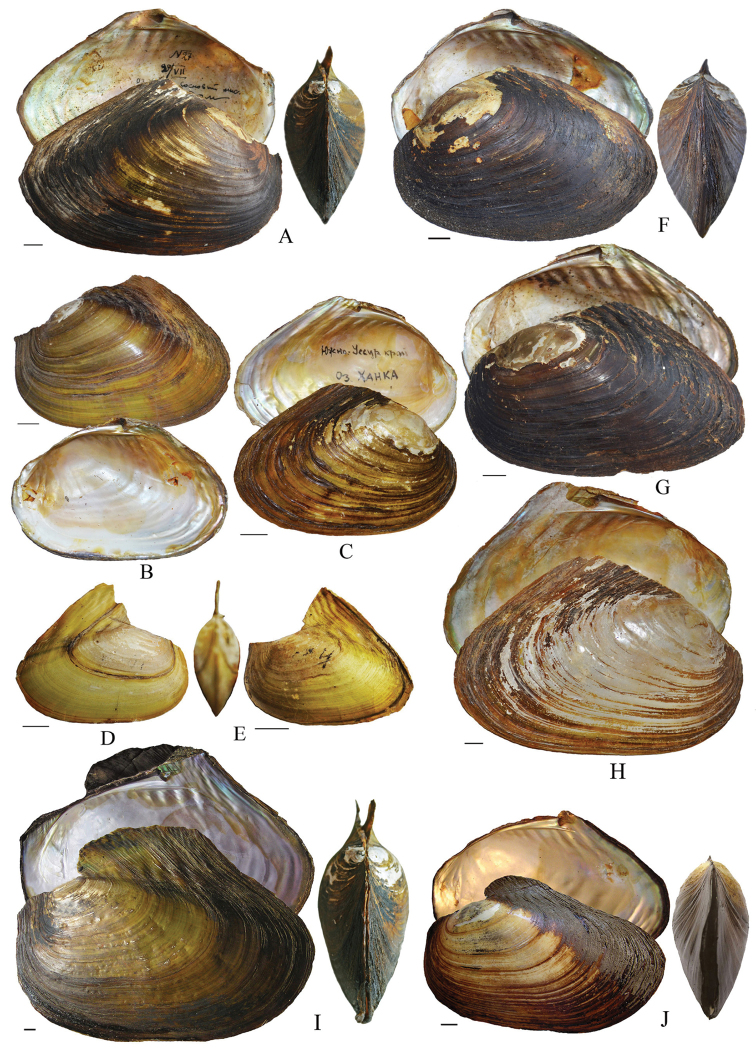
Shell morphology of *Cristaria plicata* from Eastern Russia: **A–C** *Cristaria plicata* from Khanka Lake identified by Zhadin in 1927 **D–E** *Cristaria plicata* from Khanka Lake identified by Starobogatov in 1967 **F–G** *Cristaria tuberculata* and **H** *Cristaria herculea* from Khanka Lake identified by Moskvicheva in 1971 **I** limnetic, and **J** riverine forms of Cristaria (herculea) plicata from Transbaikalia (Klishko et al. 2014).

The genus *Cristaria* exhibits high shell plasticity, common to most unionoid species. However, the basic shell morphology of *Cristaria* taxa from Russia identified by different authors as *Cristaria plicata*, *Cristaria tuberculata* and *Cristaria herculea* is very similar (Fig. 2).

**Figure 3.**
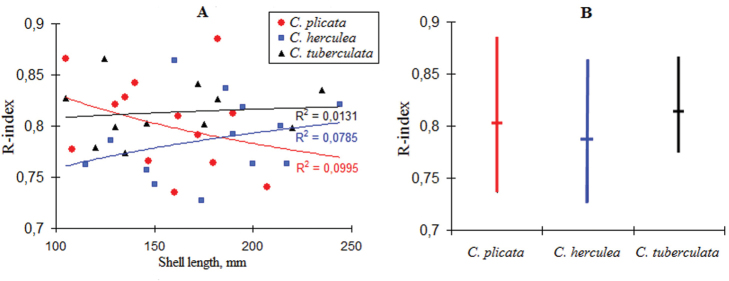
**A** Variation of the R-index with shell length **B** range and mean values of R-index for Far East Russian specimens of *Cristaria plicata*, *Cristaria herculea* and *Cristaria tuberculata*.

**Figure 4.**
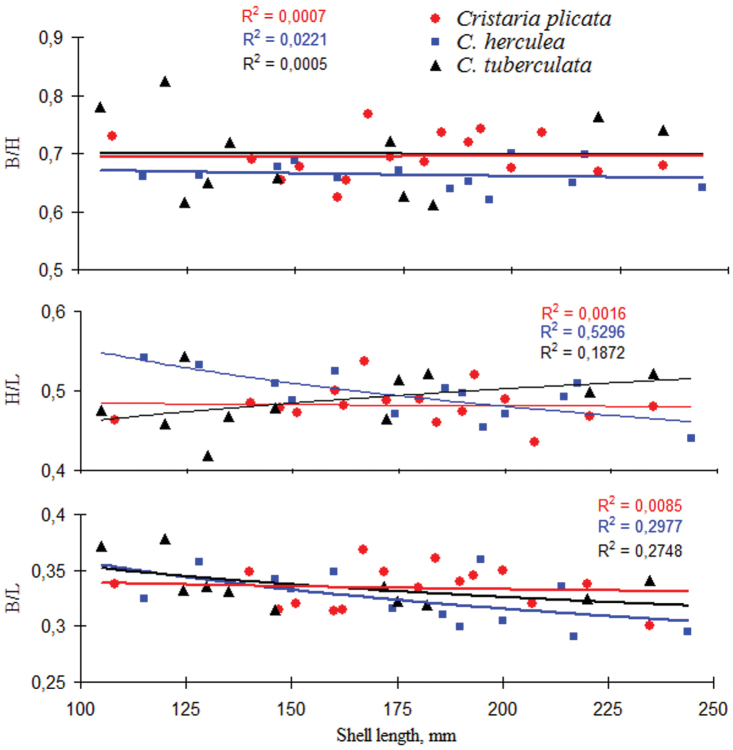
Variation of the morphometric shell indexes (B/H, H/L and B/L) with shell length in the three putative *Cristaria* taxa.

Not only do the morphometric characteristics change with the increase of shell length, but these may also vary considerably in shells from the same size. In fact, all morphometric indexes calculated in this study for *Cristaria* taxa showed wide variation (Figs 3–4). The sole character separating or distinguishing *Cristaria herculea* and *Cristaria tuberculata*, according to Starobogatov et al. (2004) (here referred as the R-index), varied from 0.727 to 0.866 with shell lengths of 100–250 mm (Fig. 3A). R-indices for *Cristaria plicata* with the same shell lengths varied from 0.735 to 0.885, completely overlapping the R-index variation ranges for *Cristaria herculea* and *Cristaria tuberculata* (Fig. 3A, B).

It should be noted that the R-index for the museum’s specimens of *Cristaria tuberculata* identified by Moskvicheva in 1971 varied between 0.801–0.813 (shell lengths: 146–175 mm), identifying these specimens as *Cristaria herculea* and not as *Cristaria tuberculata*, according to the Key of Starobogatov et al. (2004).

The values of the shell morphometric indexes (B/H, H/L and B/L) varied widely without separating any of the putative species groups (Fig. 4). In fact, there was a complete overlap in the values of the three morphological indices with shell length, for all *Cristaria* forms. Values of B/H varied in range from 0.611 to 0.825, values of H/L and B/L varied in less ranges 0.419–0.543 and 0.290–0.378, respectively but with weakly expressed trends.

**Figure 5.**
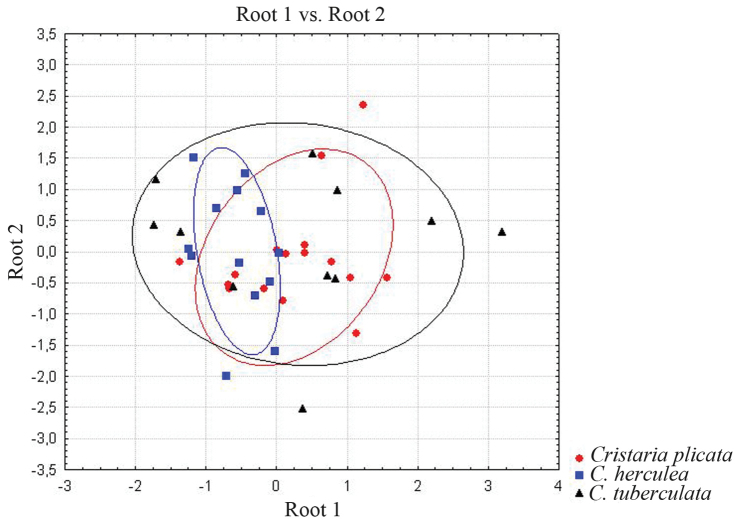
Discriminant analysis plots of *Cristaria plicata*, *Cristaria herculea* and *Cristaria tuberculata* forms from Far Eastern Russia showing the spread of the first two discriminant scores in discriminant space.

Discriminant analysis revealed no differentiation into discrete entities or species with the distribution of all points (Wilk’s l = 0.852); the discriminant plot shows a considerable overlap in all taxa of *Cristaria* (Fig. 5).

No statistically significant morphological discreteness was found between *Cristaria plicata* and forms *Cristaria herculea* and *Cristaria tuberculata* for any of the morphometric indexes used, individually or combined. The reliability of morphological discreteness assessed by Wilk’s λ values for complex indexes values, were near to 1 which indicates the absence of morphological discreteness. A discriminant analysis provided no evidence of differentiation into entities or species (Wilk’s λ = 0.852, F (6.70) = 0.974, n = 40, *p* < 0.449).

### Conservation status

*Cristaria herculea* is listed as Vulnerable in the Red Book of the Khabarovsky Krai (Voronov 2008) and as Endangered in the Red Book of the Transbaikalsky Krai (Kovaleva and Andreev 2012). *Cristaria tuberculata* is listed as Endangered in the Red Books of the Khabarovsky Krai, the Primorsky Krai and of the Russian Federation (Sokolov 2001; Kostenko 2005; Voronov 2008). It has also been recommended for registration in the forthcoming Red Book of the Russian Federation as rare and endemic, with a restricted range (Bogatov 2014). *Cristaria plicata* has been globally assessed by the IUCN as Data Deficient as further research is required on its abundance, distribution, ecology and threats (Bogan and Cummings 2011).

Until now, according to the Russian system of taxonomy, *Cristaria herculea* and *Cristaria tuberculata* were considered valid species, both being considered as threatened in regional and national Russian Red Books. Using a synthesis of morphological and genetic data, we present categorical evidence that all forms of the genus *Cristaria* inhabiting in Russia are one species, *Cristaria plicata*. This fact should be considered for future conservation measures. At the moment *Cristaria herculea* and *Cristaria tuberculata* are considered to be threatened in Eastern Russia and their populations are in decline due to anthropogenic impacts. Integrating both forms into the single species, *Cristaria plicata*, should maintain a threatened conservation status in this region. However, it is necessary to reassess the conservation status for *Cristaria plicata*, both at the regional and National (Russia) levels, using the entire distributional range and demographic trends of both previously recognized forms. Nevertheless, further research is still required on the abundance, distribution, ecology and threats to this species for a more accurate Global Red List assessment, especially in its Southern edge of distribution in China and Indochina.

### Distribution

**Figure 6.**
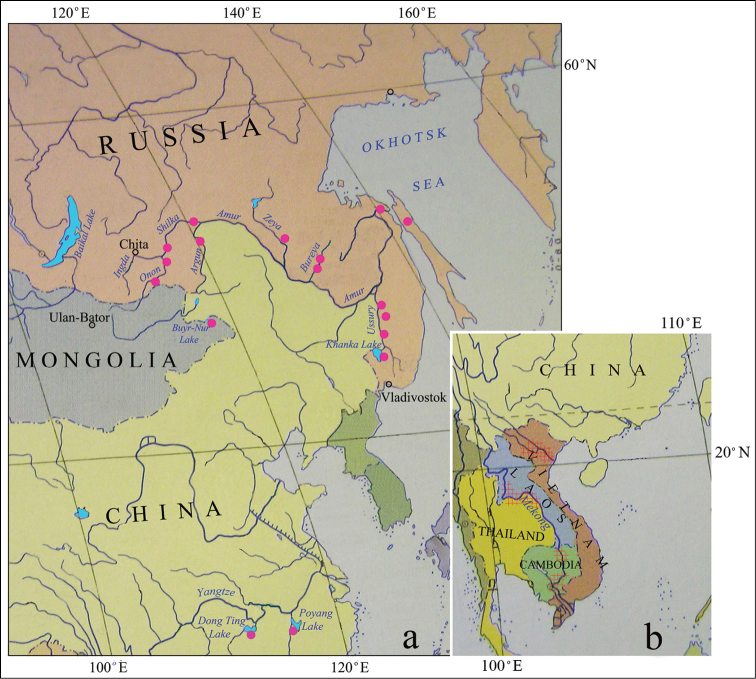
Distribution of *Cristaria plicata* in **a** Eastern Russia, Mongolia, China (pink spots) and **b** southern edge of the area in northern Vietnam, Laos, Thailand and Cambodia (pink shaded area).

*Cristaria plicata* is found across the territory of Far East Russia including the Onon, Shilka, Argun, Zeya, Bureya, Ussury river basins, the lower Amur River, the Tym River (Sakhalin Island) and Khanka Lake. It is also present in Mongolia (Buyr-Nur Lake), China (Dong Ting Lake and Poyang Lake of the Yangtze River Basin (Prozorova et al. 2005; Fig. 6a) south to Northern Vietnam, Laos, Thailand and Cambodia (Brandt 1974; Fig. 6b).

## Discussion

In a previous revision of the Far Eastern Anodontinae, and although specimens identified as *Cristaria plicata* were already present in the collection of the ZIM-SP, the genus *Cristaria* was separated into two species, *Cristaria tuberculata* and *Cristaria herculea* (Moskvicheva 1973). These forms were widely accepted by most Russian taxonomists and were separated using a comparison of conchological characters including shell convexity, the location of the umbo relative to the anterior shell margin, the position of the dorsal shell margin and the end of the wing apex (Moskvicheva 1973). Later, the use of most of these characters for classification by Russian malacologists was discontinued, but *Cristaria herculea* and *Cristaria tuberculata* were still recognized as distinct species based on shell convexity alone (*i.e.* R-index; Zatravkin and Bogatov 1987; Starobogatov et al. 2004). The present paper clearly demonstrates that there is a substantial variation and overlap of R-index values in *Cristaria herculea* and *Cristaria tuberculata* rendering them useless in delimiting these forms. Furthermore, the existence of shell forms of variable convexity can be explained by size, sex or environmental factors. In fact, it has been recognized that juvenile and middle-aged individuals have highly differentiated shell convexity values (Zatravkin and Bogatov 1987). However those differences can be obscured with age or environmental factors (Prosorova and Sayenko 2001).

Buldowsky (1935) also pointed out to the sharp morphological differences between riverine and lake forms of *Cristaria* from Ussury River and Khanka Lake. Similar differences exist between riverine and lake forms of *Cristaria* from Transbaikalia (Fig. 2I, J). Studies carried out by Zieritz and Aldridge (2011) on another Anodontine species show that female *Anodonta anatina* specimens are generally more inflated than males, in order to increase the volume of the branchial chambers for glochidial brooding. Unfortunately, published studies on the *Cristaria* from Far East Russia dealt only with shells without sexing the individuals. Thus, while the present study has shown that shell convexity in *Cristaria* can vary with age, a possibility remains that it also might vary with sex. This may explain the previous conchological distinctions between the more or less convex shell shapes of *Cristaria tuberculata* and *Cristaria herculea* as a simple description of specimens of distinct sizes or as males and females of the same species, *Cristaria plicata*.

## Supplementary materials

Supplementary material 1 References used to determine Cristaria taxa distribution Data type: **references list** Explanation note: References used to determine *Cristaria* taxa distribution. * - Museum of Zoological Institute, Russian Academy of Sciences, St.-Petersburg; ** - unpublished. This dataset is made available under the Open Database License (http://opendatacommons.org/licenses/odbl/1.0/). The Open Database License (ODbL) is a license agreement intended to allow users to freely share, modify, and use this Dataset while maintaining this same freedom for others, provided that the original source and author(s) are credited.Olga K. Klishko, Manuel Lopes-Lima, Elsa Froufe, Arthur E. Bogan, Vera Y. Abakumova
