# Supplementary material for: Systematics and distribution of Cristaria plicata (Bivalvia, Unionidae) from the Russian Far East
Source: Zookeys. 2016 Apr 12;(580):13–27. doi: 10.3897/zookeys.580.7588 (PMC4829948; doi:10.3897/zookeys.580.7588)
Supplement: Supplementary material 1 — References used to determine Cristaria taxa distribution [file zookeys-580-013-s001.docx]

**Supplementary table 1.** References used for *Cristaria* taxa distribution. * - Museum of Zoological Institute, Russian Academy of Sciences, St.-Petersburg; ** - unpublished.

| Taxon | Location | Reference |
| --- | --- | --- |
| *C. plicata*  (Leach, 1815) | Basins of Amur River and Ussury River, Khanka Lake. | Zhadin (1938, 1952) |
| *C. plicata*  (Leach, 1815) | Khanka Lake, different localities* | *Collector Kobyakova and Baturina (1931), identified by Zhadin in 1927 and Starobogatov in 1967. |
| *C. plicata*  (Leach, 1815) | Dong Ting Lake and Poyang Lake of Yangtze River Basin in south of China; basin of Mekong River of Indochina.  Transbaikalia: Onon, Shilka and Nercha rivers, Kharanorsky reservoir; Buyr-Nur Lake, Mongolia | Prozorova et al. (2005)  Klishko et al. (2014) |
| *C. plicata*  (Leach, 1815) | Lower Yangtze and Zhejiang in China  North Korea (Unknown) | Jia and Li**; Zhang et al*,***; Jiang et al. (2010)  Park et al.*,***; Lee et al. (2012) |
| *C. herculea*  (*Anodonta herculea*) Middendorff, 1847 | Amur and Ussury riverbasins, Khanka Lake. | Middendorff (1847)  Middendorff (1851) |
| *C. herculea* (Middendorff, 1847) | Basins of Amur River and Khanka Lake, Buyr-Nur Lake*; Zeya River;  Dolgoe Lake, Listvyanoe Lake of Bureya River Basin; Tym River Sakhalin Island; Transbaikalia: Onon, Nercha, Shilka, Argun rivers; Charanorsky reservoir | Zatravkin (1983)  Zatravkin and Bogatov (1987)  Prozorova and Sayenko (2001)  Sayenko and Balan (2009)  Starobogatov et al. (2004)  Voronov (2008)  Kovaleva & Andreev (2012) |
| *C. tuberculata* Schumacher, 1817 | Ussury River, Mandzhurka River, Khanka Lake; basins of Khanka Lake and Ussury River;  Khanka Lake* | Moskvicheva (1973)  Zatravkin and Bogatov (1987)  Prozorova and Sayenko (2001)  Starobogatov et al. (2004)  Sokolov (2001)  Kostenko (2005)  *Collection expedition of the Far East Branch Academy of Sciences USSR, identified by Moskvicheva in 1971. |

**References**

Jiang WP, Li JL, Zheng RL, Wang GL (2010) Analysis of complete mitochondrial genome of *Cristaria plicata*. Yi Chuan 32: 153‒162.

Klishko OK, Lopes-Lima M, Froufe E, Bogan AE (2014) Are *Cristaria herculea* (Middendorff, 1847) and *Cristaria plicata* (Leach, 1815) (Bivalvia: Unionidae) separate species? Zookeys 438: 1‒15.

Kostenko VA (2005) Red Book of the Primorsky Krai: Animals. AVK Apelsin, Vladivostok, 408 pp. [in Russian]

Kovaleva TC, Andreev AV (2012) Red Book of the Transbaikalsky Krai: Animals. Novosibirsky Publish House, Novosibirsk, 345 pp. [in Russian]

Lee JH, Choi EH, Kim SK, Ryu SH, Hwang UW (2012) Mitochondrial genome of the cockscomb pearl mussel *Cristaria plicata* (Bivalvia, Unionoida, Unionidae). Mitochondrial DNA 23: 39‒41. Doi: 10.3109/19401736.2011.643882

Middendorff A (1847) Sur un envoi adressé à l 'Academie par M. Sensinov de Nertchinsk et sur une novelle espece d'*Anodonta*. Bulletin de la Classe Physico-Mathématique de l’Académie Impériale des Sciences de Saint-Pétersbourg 6: 302‒304.

Middendorff A (1851) Mollusken. In: Reise in den äussersten Norden und Osten Sibiriens während der Jahre 1843 und 1844. St.-Petersbourg, Vol. 2, 163‒164 and Vol. 3, 278‒279.

Moskvicheva IM (1973) Molluscs of subfamily Anodontinae (Bivalvia, Unionidae) of Amur basin and Primorye territory. Zoological Journal 52: 822‒834. [in Russian]

Prozorova LA, Sayenko EM (2001) To the biology of the Anodontinae genus *Cristaria* (Bivalvia, Unionidae). Ruthenica 11: 33‒36. [in Russian]

Prozorova LA, Sayenko EM, Bogatov VV, Wu M, Liu YY (2005) Bivalves of the Yangtze River drainage. Bulletin of the Russian Far East Malacological Society 9: 46‒58. [in Russian]

Sayenko EM, Balan IV (2009) New data on large bivalves (Margaritiferidae, Unionidae) of the Khingansky Reserve and adjacent areas (Amurskaya Territory). Bulletin of the Russian Far East Malacological Society 13: 63‒69. [in Russian]

Sokolov VE (2001) Red Book of the Russian Federation: Animals. AST Astrel, Moscow, 862 pp. [in Russian]

Starobogatov YI, Prozorova LA, Bogatov VV, Sayenko EM, Khlebovich VV, Chernyshev AV (2004) Key to freshwater invertebrates of Russia and adjacent lands. Vol. 6. Molluscs, Polychaetes, Nemerteans. Nauka, St. Petersburg, 528 pp. [in Russian]

Voronov BA (2008) Red Book of the Khabarovsky Krai: Animals. Priamurskiye Vedomosti Publish House, Khabarovsk, 632 pp. [in Russian]

Zatravkin MN, 1983. Unionoidea of the USSR fauna and their role as alternate host of trematodes: Molluscs. Taxonomy, ecology and distribution conformity. Nauka, Leningrad 7, 40‒44. [in Russian]

Zatravkin MN, Bogatov VV (1987) Large bivalve molluscs of the fresh and brackish waters of the USSR Far East. DVO AN USSR, Vladivostok, 152 pp. [in Russian]

Zhadin VI (1938) Family Unionidae. Fauna of USSR. Mollusca, Vol. 4. Moscow-Leningrad, USSR. [in Russian]

Zhadin VI (1952) Mollusks of fresh and brackish waters of the USSR. Identification guides on the USSR fauna. Zoological Institute of the USSR Academy of Sciences 46: 1‒376.
